# Chronic physical conditions, multimorbidity and physical activity across 46 low- and middle-income countries

**DOI:** 10.1186/s12966-017-0463-5

**Published:** 2017-01-18

**Authors:** Davy Vancampfort, Ai Koyanagi, Philip B. Ward, Simon Rosenbaum, Felipe B. Schuch, James Mugisha, Justin Richards, Joseph Firth, Brendon Stubbs

**Affiliations:** 1 Department of Rehabilitation Sciences, KU Leuven, Tervuursevest 101, Leuven, 3001 Belgium; 2 KU Leuven, University Psychiatric Center KU Leuven, Leuvensesteenweg 517, Kortenberg, 3070 Belgium; 3 Research and Development Unit, Parc Sanitari Sant Joan de Déu, Universitat de Barcelona, Fundació Sant Joan de Déu, Dr. Antoni Pujadas, 42, Sant Boi de Llobregat, Barcelona, 0883 Spain; 4 Instituto de Salud Carlos III, Centro de Investigación Biomédica en Red de Salud Mental, CIBERSAM, Monforte de Lemos 3-5 Pabellón 11, Madrid, 28029 Spain; 5 School of Psychiatry, UNSW, Sydney, Australia; 6 Schizophrenia Research Institute, Ingham Institute of Applied Medical Research, Liverpool, NSW Australia; 7 Exercise Physiology Department, School of Medical Sciences, UNSW Australia, Sydney, Australia; 8 Hospital de Clínicas de Porto Alegre, Porto Alegre, Brazil; 9 Programa de Pós Graduação em Ciências Médicas: Psiquiatria, Universidade Federal do Rio Grande do Sul, Porto Alegre, Brazil; 10 Kyambogo University, Kampala, Uganda; 11 Butabika National Referral and Mental Health Hospital, Kampala, Uganda; 12 School of Public Health & Charles Perkins Centre, University of Sydney, Sydney, Australia; 13 School of Health Sciences, Division of Psychology & Mental Health, University of Manchester, Manchester, UK; 14 Physiotherapy Department, South London and Maudsley NHS Foundation Trust, Denmark Hill, London, SE5 8AZ UK; 15 Health Service and Population Research Department, Institute of Psychiatry, Psychology and Neuroscience, King’s College London, Box SE5 8AF, De Crespigny Park, London, UK

**Keywords:** Multimorbidity, Pain, Mobility limitation, Depression, Sleep, Physical activity, Arthritis, Angina pectoris, Diabetes mellitus

## Abstract

**Background:**

There are no nationally representative population-based studies investigating the relationship between physical activity, chronic conditions and multimorbidity (i.e., two or more chronic conditions) in low- and middle-income countries (LMICs), and studies on a multi-national level are lacking. This is an important research gap, given the rapid increase in the prevalence of chronic diseases associated with lifestyle changes in these countries. This cross-sectional study aimed to assess the association between chronic conditions, multimorbidity and low physical activity (PA) among community-dwelling adults in 46 LMICs, and explore the mediators of these relationships.

**Methods:**

World Health Survey data included 228,024 adults aged ≥18 years from 46 LMICs. PA was assessed by the International Physical Activity Questionnaire (IPAQ). Nine chronic physical conditions (chronic back pain, angina, arthritis, asthma, diabetes, hearing problems, tuberculosis, visual impairment and edentulism) were assessed. Multivariable logistic regression and mediation analyses were used to assess the association between chronic conditions or multimorbidity and low PA.

**Results:**

Overall, in the multivariable analysis, arthritis (OR = 1.12), asthma (1.19), diabetes (OR = 1.33), edentulism (OR = 1.46), hearing problems (OR = 1.90), tuberculosis (OR = 1.24), visual impairment (OR = 2.29), multimorbidity (OR = 1.31; 95% CI = 1.21–1.42) were significantly associated with low PA. More significant associations were observed in individuals aged ≥50 years. In older adults, depression mediated between 5.1% (visual impairment) to 23.5% (angina) of the association between a chronic condition and low PA. Mobility difficulties explained more than 25% of the association for seven of the eight chronic conditions. Pain was a strong mediator for angina (65.9%) and arthritis (64.9%), while sleep problems mediated up to 43.7% (angina) of the association.

**Conclusions:**

In LMICs, those with chronic conditions and multimorbidity are significantly less physically active (especially older adults). Research on the efficacy and effectiveness of PA in the management of chronic diseases in LMICs is urgently needed. Targeted promotion of physical activity to populations in LMICs experiencing chronic conditions may ameliorate associated depression, mobility difficulties and pain that are themselves important barriers for initiating or adopting an active lifestyle.

**Electronic supplementary material:**

The online version of this article (doi:10.1186/s12966-017-0463-5) contains supplementary material, which is available to authorized users.

## Background

While the average life expectancy is increasing worldwide, the number of years lived with disability with various chronic conditions is also rising [1, 2]. Of particular concern is the increasing global burden of angina [3], arthritis [4], asthma [5], chronic back pain [6], diabetes [7], oral diseases, such as edentulism [8], hearing problems [9], tuberculosis [10], and visual impairments [11], mainly due to population growth and aging of the worldwide population. There is also an increasing recognition that in the years to come, this disease burden and the loss of economic output associated with chronic diseases will be greatest in low- and middle-income countries (LMICs) [12].

Recently, more research has noted the burden of multimorbidity (i.e., two or more chronic conditions) [13]. In a meta-analysis [14] of 70,057,611 primary care patients in 12 countries, the prevalence of multimorbidity ranged from 12.9 to 95.1%. The prevalence of multimorbidity is increasing, mainly due to the growing incidence of chronic conditions and increasing life-expectancy [15], and it is undoubtedly one of the most significant challenges faced by global health care providers [16]. Multimorbidity is associated with a lower quality of life [17], increased health-care utilization and costs [18], and ultimately, higher risk for premature mortality [19]. The worldwide evolving disease burden [1], along with a growing understanding of multimorbidity and its risk factors [20], necessitates a continuum of care.

Within the multifaceted care of individuals with chronic disease and multimorbidity, the promotion of physical activity is extensively supported in the published literature [21]. Regular physical activity contributes to the primary and secondary prevention of a wide range of chronic diseases [21], improves quality of life [22] and is associated with reduced risk of premature death [23]. However, to date, most of the research investigating associations between physical activity, chronic diseases and multimorbidity has focused on high-income countries. For example, in a Spanish study [24] involving 22,190 adults, an inverse association was found between multimorbidity and levels of physical activity participation in the youngest and oldest age groups. In addition, both low self-rated health status and functional limitations were related to lower physical activity in most of the examined population groups. In an English nationally representative cohort of people aged ≥50 years (*n* = 15,688) [25], compared to the physically inactive group, the odds ratio (OR) for multimorbidity was 0.84 (95% confidence interval (CI) = 0.78–0.91) in the mild, 0.61 (95% CI = 0.56–0.66) in the moderate, and 0.45 (95% CI = 0.41–0.49) in the vigorous physical activity groups.

However, to the best of our knowledge, there are no nationally representative population-based studies investigating the associations between physical activity behavior, chronic conditions and multimorbidity in LMICs. Moreover, to the best of our knowledge there are no studies investigating physical activity and multimorbidity on a multi-national level. This is an important research gap given the rapid increase in chronic diseases in these countries, mainly due to changes in lifestyle [1]. Furthermore, the association between chronic conditions or multimorbidity on physical activity behavior may differ in LMICs due to different disease profiles [26], suboptimal treatment of chronic conditions [27, 28], differences in knowledge regarding the benefits of physical activity [29], or other environmental factors such as work conditions [30]. In addition, at the population level, there is a paucity of information on factors that might influence the relationship between physical activity, chronic diseases and multimorbidity. Such information could guide the design and delivery of targeted interventions.

Given the aforementioned gaps within the literature, we aimed to assess the association between chronic conditions or multimorbidity and low physical activity (i.e., not achieving international physical activity recommendations) among community-dwelling adults in 46 LMICs, and to assess the factors that might influence this relationship. We hypothesize that low physical activity is associated with the presence of chronic conditions and multimorbidity.

## Methods

### Settings and protocol

The World Health Survey (WHS) was a cross-sectional study undertaken in 2002–2004 in 70 countries worldwide. Single-stage random sampling and stratified multi-stage random cluster sampling were conducted in 10 and 60 countries respectively. The details of the survey have been provided elsewhere (http://www.who.int/healthinfo/survey/en/). Briefly, all those aged ≥18 years with a valid home address were eligible to participate. Each member of the household had equal probability of being selected with the use of Kish tables. The data were collected in all countries using the same set of questionnaires with some countries however using a shorter version. The individual response rate ranged from 63% (Israel) to 99% (Philippines) [31]. Ethical approval was obtained from ethical boards at each study site. Sampling weights were generated to adjust for non-response and the population distribution reported by the United Nations Statistical Division. Informed consent was obtained from all participants.

### Physical activity

In order to assess if participants achieved the recommended physical activity levels of 150 min of moderate to vigorous physical activity per week [32], we used items from the International Physical Activity Questionnaire. Specifically, participants were asked how much over the past week on average they engaged in moderate and vigorous physical activity. Those scoring ≥150 min were classified as meeting the recommended guidelines and those scoring <150 min (low physical activity) were classified as not meeting the recommended guidelines.

### Physical health conditions

A total of nine physical conditions were assessed, representing all physical conditions available in the WHS. Arthritis, asthma and diabetes were based on self-reported lifetime diagnosis. For angina, in addition to a self-reported diagnosis, a symptom-based diagnosis based on the Rose questionnaire was also used [33]. Chronic back pain was defined as having had back pain (including disc problems) every day during the last 30 days. Visual impairment was defined as having extreme difficulty in seeing and recognizing a person that the participant knows across the road (i.e., from a distance about 20 m) [34]. A validity study showed that this response generally corresponds to World Health Organization definitions of visual impairment [34]. The participant was considered to have hearing problems if the interviewer observed this condition at the end of the survey. Edentulism was assessed by the question “Have you lost all your natural teeth?” Those who responded affirmatively were considered to have edentulism. Finally, a tuberculosis diagnosis was based on past 12-month symptoms and was defined as: 1) having had a cough that lasted for three weeks or longer; and 2) having had blood in phlegm or coughed up blood [35]. In line with a previous publication using the same dataset [36], we calculated the total number of these conditions while allowing for one missing variable in order to retain a larger sample size. Multimorbidity was defined as having at least two of the assessed chronic conditions.

### Health status and depression

Participants’ health status was evaluated with six health-related questions pertaining to three health domains including (a) mobility; (b) pain and discomfort; (c) sleep and energy (Additional file 1). These domains have been used as indicators of functional health status in prior studies utilizing the WHS dataset [37–39]. Each domain consists of two questions that assessed health function in the past 30 days. Each item was scored on a five-point scale ranging from ‘none’ to ‘extreme/cannot do’. For each separate domain, we used a factor analysis to obtain a factor score which was later converted to scores ranging from 0 to 100 [37, 39] with higher values representing worse health function. In order to determine the presence of depression, the DSM-IV algorithm was used, based on the duration and persistence of depressive symptoms in the previous 12 months [40].

### Control variables

The control variables included sex, age, highest educational level achieved (no formal education, primary education, secondary or high school completed, or tertiary education completed) and wealth. Principal component analysis based on 15–20 assets was performed to establish country-wise wealth quintiles.

### Statistical analysis

Data from 69 countries were publically available. Of these countries, 10 countries (Austria, Belgium, Denmark, Germany, Greece, Guatemala, Italy, Netherlands, Slovenia, UK) were deleted as sampling information was missing. Furthermore, 10 high-income countries (Finland, France, Ireland, Israel, Luxembourg, Norway, Portugal, Sweden, Spain, United Arab Emirates) were omitted as the focus of the study was on LMICs. Of the remaining LMICs, Morocco and Latvia were not included as they lacked information on physical activity, and Turkey was also excluded due to lack of several variables pertaining to the analysis. Thus, a total of 46 countries, which were all LMICs according to the World Bank classification in 2003, were included in the analysis [41] (see Table 1). We stratified the analyses by age (18–34, 35–49, 50–64, ≥65 years) as chronic conditions are known to be much more prevalent in the older population. Differences in sample characteristics by age group were evaluated by Chi-squared tests. Across all countries, we conducted multivariable logistic regression analysis to assess the association between chronic conditions (angina, arthritis, asthma, chronic back pain, diabetes, edentulism, hearing problem, tuberculosis, visual impairment) or multimorbidity (exposure variables) and low physical activity (outcome variable) while adjusting for age, sex, wealth, education and country. Each chronic condition and multimorbidity were included separately in the models. Furthermore, based on the results of these analyses, we conducted mediational analysis to evaluate underlying factors that may explain the link between chronic conditions or multimorbidity and low physical activities among those aged ≥50 years. We only included the older age group for this analysis as most of the significant association between chronic conditions (or multimorbidity) and low physical activity were only observed in the older age groups. We did not conduct this analysis for chronic back pain as this condition was not significantly associated with low physical activity in either of the older age groups.

**Table 1:** Prevalence of low physical activity by country and age groups

Country	N	Overall		Age 18–34 years		Age 35–49 years		Age 50–64 years		Age ≥65 years	
Bangladesh	5552	22.3	[20.0,24.7]	18.9	[16.2,21.9]	16.6	[13.9,19.8]	31.5	[27.7,35.6]	61.3	[54.3,67.9]
Bosnia Herzegovina	1028	25.7	[22.0,29.9]	17.2	[12.2,23.7]	13.2	[8.3,20.4]	38.1	[27.6,49.9]	58.9	[48.2,68.8]
Brazil	5000	33.6	[31.5,35.7]	28.4	[25.9,31.1]	30.2	[27.3,33.4]	39.5	[35.4,43.7]	61.2	[56.7,65.6]
Burkina Faso	4824	14.4	[12.6,16.4]	9.2	[7.6,11.2]	12.1	[9.5,15.2]	25.8	[21.0,31.4]	56.4	[48.5,64.1]
Chad	4644	39.0	[31.6,46.9]	35.5	[28.3,43.6]	40.8	[32.3,49.8]	45.1	[36.2,54.2]	51.5	[40.7,62.2]
China	3993	39.3	[31.6,47.6]	36.9	[26.7,48.5]	30.8	[23.0,39.9]	37.2	[30.3,44.7]	69.4	[60.9,76.7]
Comoros	1759	9.6	[7.5,12.2]	6.4	[4.3,9.5]	4.5	[2.4,8.4]	10.5	[7.1,15.4]	28.2	[20.9,36.7]
Croatia	990	26.4	[23.3,29.7]	17.0	[11.7,24.1]	17.5	[12.7,23.5]	28.8	[22.8,35.7]	45.0	[37.6,52.5]
Czech Republic	935	35.6	[30.6,41.0]	23.3	[18.1,29.5]	29.7	[20.8,40.3]	35.9	[27.0,45.8]	66.8	[55.4,76.5]
Dominican Republic	4534	58.9	[56.1,61.6]	55.8	[51.8,59.7]	58.3	[53.9,62.7]	59.6	[53.9,65.0]	78.6	[72.4,83.7]
Ecuador	4654	39.2	[33.5,45.2]	38.0	[31.3,45.1]	37.9	[30.8,45.6]	37.0	[27.2,48.0]	60.9	[46.5,73.6]
Estonia	1011	20.0	[16.7,23.7]	13.2	[9.7,17.7]	16.9	[12.6,22.2]	17.7	[13.2,23.2]	36.9	[29.0,45.5]
Ethiopia	4937	5.4	[4.2,7.0]	4.5	[3.1,6.4]	3.7	[2.6,5.4]	7.0	[4.5,10.6]	24.7	[15.4,37.2]
Georgia	2755	32.1	[26.5,38.2]	29.5	[22.7,37.3]	28.1	[21.4,35.9]	24.6	[19.5,30.5]	51.3	[44.3,58.2]
Ghana	3935	29.5	[27.2,32.0]	27.7	[24.5,31.2]	26.9	[23.7,30.3]	31.6	[27.1,36.5]	51.8	[45.5,58.1]
Hungary	1419	18.6	[15.9,21.6]	14.6	[11.1,19.0]	14.3	[10.2,19.7]	14.6	[10.8,19.4]	35.8	[29.2,42.9]
India	9988	18.9	[15.5,22.9]	14.6	[10.9,19.3]	15.7	[13.0,18.7]	22.3	[18.0,27.4]	45.6	[36.2,55.4]
Ivory Coast	3184	36.4	[32.7,40.2]	36.6	[32.3,41.0]	30.8	[25.8,36.3]	37.4	[30.6,44.8]	61.0	[49.8,71.2]
Kazakhstan	4496	38.6	[31.0,46.8]	33.8	[27.6,40.7]	32.7	[25.6,40.8]	46.0	[35.2,57.2]	64.0	[48.9,76.8]
Kenya	4412	12.4	[9.8,15.5]	11.4	[8.5,15.1]	13.1	[9.5,17.8]	12.7	[7.9,19.9]	22.4	[15.7,30.9]
Laos	4888	26.3	[24.3,28.4]	21.6	[19.1,24.2]	22.3	[19.9,24.9]	34.8	[31.0,38.9]	62.3	[55.6,68.6]
Malawi	5289	16.5	[14.8,18.4]	16.2	[14.1,18.5]	14.2	[12.4,16.2]	15.8	[11.4,21.4]	27.9	[20.9,36.1]
Malaysia	6040	31.8	[30.1,33.5]	29.7	[27.4,32.2]	26.1	[23.6,28.8]	37.7	[34.2,41.4]	56.6	[51.1,61.9]
Mali	3856	18.4	[15.6,21.5]	14.8	[11.5,18.9]	16.8	[12.7,21.9]	23.3	[17.1,30.9]	56.3	[44.0,67.9]
Mauritania	3795	81.8	[78.2,85.0]	79.7	[75.0,83.7]	79.6	[74.8,83.7]	88.5	[84.4,91.7]	93.7	[88.0,96.8]
Mauritius	3888	29.5	[26.0,33.3]	25.5	[21.4,29.9]	23.1	[19.2,27.4]	35.2	[30.1,40.7]	60.7	[53.6,67.4]
Mexico	38,745	31.5	[30.1,32.9]	28.8	[27.3,30.4]	29.1	[27.4,30.9]	34.5	[32.5,36.6]	49.9	[47.4,52.3]
Myanmar	5886	24.1	[20.7,27.8]	19.5	[16.1,23.5]	19.3	[15.8,23.3]	29.9	[25.2,35.0]	58.4	[52.3,64.2]
Namibia	4246	48.7	[46.3,51.0]	43.3	[40.2,46.5]	50.6	[46.3,54.8]	56.5	[50.7,62.2]	63.8	[56.8,70.3]
Nepal	8686	14.7	[13.7,15.8]	11.6	[10.3,13.1]	11.7	[10.3,13.3]	17.6	[15.2,20.3]	45.5	[41.4,49.6]
Pakistan	6377	51.1	[48.9,53.3]	46.0	[43.3,48.8]	48.4	[44.7,52.1]	60.3	[55.7,64.7]	78.9	[72.2,84.3]
Paraguay	5142	25.6	[23.9,27.3]	25.5	[23.3,27.8]	21.1	[18.7,23.7]	25.7	[22.3,29.4]	46.2	[40.6,52.0]
Philippines	10,076	11.9	[10.7,13.3]	11.0	[9.5,12.6]	8.5	[7.2,10.0]	13.6	[11.4,16.1]	30.9	[26.5,35.8]
Republic of Congo	2492	43.7	[34.2,53.8]	39.7	[29.8,50.5]	47.7	[37.8,57.8]	48.2	[24.8,72.4]	66.5	[47.9,81.0]
Russia	4421	30.6	[27.3,34.1]	22.3	[15.7,30.6]	20.3	[15.6,26.1]	32.9	[26.0,40.6]	45.1	[40.1,50.3]
Senegal	3180	31.5	[27.6,35.8]	25.0	[20.6,29.9]	32.3	[26.0,39.4]	50.3	[41.0,59.6]	63.3	[51.9,73.4]
Slovakia	2492	25.1	[20.0,31.0]	14.5	[10.7,19.5]	32.2	[23.6,42.2]	27.8	[13.0,50.0]	32.7	[16.3,54.8]
South Africa	2350	66.6	[62.5,70.5]	61.9	[56.9,66.6]	67.6	[62.6,72.2]	75.1	[67.4,81.5]	80.1	[71.4,86.6]
Sri Lanka	6732	22.3	[19.4,25.6]	20.0	[16.6,23.9]	15.6	[13.0,18.7]	23.8	[18.4,30.3]	50.2	[44.0,56.4]
Swaziland	3107	48.7	[44.4,53.1]	46.6	[40.9,52.3]	48.2	[42.1,54.3]	50.1	[40.3,59.8]	65.1	[52.3,76.0]
Tunisia	5068	46.9	[43.4,50.5]	42.6	[38.7,46.6]	43.1	[38.9,47.4]	52.4	[46.9,57.9]	74.0	[68.4,79.0]
Ukraine	2847	20.6	[17.0,24.6]	16.2	[11.8,21.8]	15.1	[11.7,19.4]	20.6	[15.5,26.9]	36.7	[29.2,44.9]
Uruguay	2979	63.9	[55.6,71.3]	53.9	[45.2,62.4]	58.7	[53.8,63.5]	69.3	[59.5,77.6]	84.6	[72.8,91.9]
Vietnam	3492	17.9	[14.0,22.5]	11.6	[8.5,15.6]	13.9	[10.2,18.7]	26.0	[19.0,34.4]	50.6	[41.0,60.2]
Zambia	3808	25.6	[22.9,28.5]	23.6	[20.4,27.0]	24.7	[20.8,29.1]	26.8	[21.5,33.0]	45.5	[36.5,54.7]
Zimbabwe	4092	20.3	[18.5,22.3]	17.4	[15.2,19.8]	19.9	[15.9,24.6]	22.7	[18.5,27.5]	43.9	[35.4,52.7]

Data are unweighted N and % [95% confidence interval]

The total amount of moderate to vigorous physical activity over the last week was calculated and those scoring <150 min were considered to have low physical activity

Given that depression, mobility difficulties, pain/discomfort and sleep problems may be linked with chronic conditions as part of the symptomatology per se or the consequences of the symptoms [42], we investigated the mediating effect of these factors with the use of Karlson-Holm-Breen command in Stata [43]. This method can be applied to logistic regression models and decomposes the total effect into direct and indirect effects. Using this method, the mediated percentage (percentage of the main association explained by the mediator) can also be calculated. Each potential mediator was included in the models separately, and the models were adjusted for age, sex, education, wealth and country. For all analyses, adjustment for country was done by including dummy variables for each country as in previous WHS publications [37, 44]. The percentage of missing values for all the variables used in this study were <10% with the exception of the number of chronic conditions (10.9%), edentulism (12.2%), and tuberculosis (15.0%). Complete-case analysis was done. The sample weighting and the complex study design were taken into account in all analyses. Results from the logistic regression models are presented as odds ratios (ORs) with 95% confidence intervals (CIs). The level of statistical significance was set at *P* < 0.05. The statistical analysis was performed with Stata 14.1 (Stata Corp LP, College station, Texas).

## Results

The final sample consisted of 228,024 individuals aged ≥18 years. Of these individuals, 97,841 (47.9%), 68,657 (27.5%), 37,688 (16.0%), and 23,838 (8.6%) were aged 18–34, 35–49, 50–64 and ≥65 years respectively. The prevalence of low physical activity in the overall sample was 29.2% (95% CI = 28.3–30.0%). The corresponding figures by age group were: 18–34 years [25.1% (24.2–26.1%)]; 35–49 years [25.5% (24.6–26.5%)]; 50–64 years [34.9% (33.5–36.3%)]; and ≥65 years [53.3% (51.4–55.2%)]. The overall prevalence of low physical activity ranged from 5.4% (Ethiopia) to 81.8% (Mauritania) (Table 1).

More than half of the population also engaged in low physical activities in South Africa (66.6%), Uruguay (63.9%), the Dominican Republic (58.9%), and Pakistan (51.1%). Older individuals were significantly more likely to be females, have lower education and wealth, chronic conditions and a higher number of chronic conditions (Table 2).

**Table 2:** Sample characteristics (overall and by age group)

Characteristic		Total	Age 18–34 years	Age 35–49 years	Age 50–64 years	Age ≥65 years	*P*-value
Age (years)	Mean (SD)	38.4 (16.1)	25.0 (4.5)	41.1 (4.4)	55.7 (4.4)	72.2 (7.0)	
Sex	Female	50.8	49.5	50.3	52.6	56.2	<0.0001
Education	No formal	26.1	19.3	27.5	36.5	40.1	<0.0001
	≤Primary	31.0	31.7	31.0	29.5	30.7	
	Secondary completed	33.7	39.8	31.6	24.8	22.9	
	Tertiary completed	9.2	9.3	10.0	9.3	6.3	
Wealth	Poorest	20.1	19.3	19.6	19.8	27.3	<0.0001
	Poorer	20.0	19.3	19.9	20.6	22.5	
	Middle	19.9	20.3	19.6	19.5	19.5	
	Richer	20.0	20.6	20.2	19.8	16.1	
	Richest	20.0	20.5	20.7	20.4	14.6	
Chronic conditions	Angina	14.6	8.9	14.3	22.2	32.5	<0.0001
	Arthritis	13.0	6.0	12.6	24.1	33.3	<0.0001
	Asthma	5.2	3.9	4.8	7.0	9.6	<0.0001
	Chronic back pain	6.6	3.2	6.8	10.9	16.6	<0.0001
	Diabetes	3.0	0.7	2.6	7.3	9.0	<0.0001
	Edentulism	5.9	1.5	3.5	10.9	28.9	<0.0001
	Hearing problem	3.4	1.0	1.8	4.5	20.0	<0.0001
	Tuberculosis	1.7	1.3	1.5	2.5	2.6	<0.0001
	Visual impairment	1.3	0.4	0.9	2.2	6.4	<0.0001
Number of chronic conditions	0	65.3	78.8	65.7	45.7	25.1	<0.0001
	1	21.7	16.8	23.5	29.8	28.2	
	2	8.5	3.6	8.2	15.6	23.5	
	3	3.1	0.7	2.1	6.4	14.0	
	≥4	1.4	0.1	0.5	2.5	9.2	

Data are weighted column % unless otherwise stated

The differences in sample characteristics between age groups were tested by Chi-squared tests

**Fig. 1:**
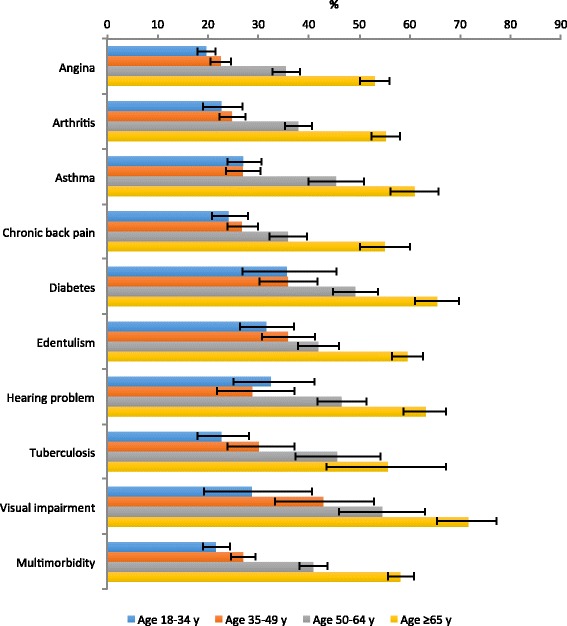
Prevalence of low physical activity in individuals with each chronic condition or multimorbidity by age group

For most chronic conditions, the prevalence of low physical activity increased in a linear fashion with increasing age. There was a particularly high prevalence of low physical activity among those with visual impairment and diabetes especially among the older population (Fig. 1).

The results of the multivariable logistic regression analysis assessing the association between chronic conditions or multimorbidity and low physical activity are presented in Table 3. In the overall sample, arthritis, asthma, diabetes, edentulism, hearing problems, tuberculosis, visual impairment and multimorbidity were significantly associated with low physical activity. When the analysis was stratified by age groups, most significant associations only existed in the older age groups (i.e., ≥50 years). For example, the ORs (95% CIs) for the following conditions among those aged 50–64 years were: angina 1.17 (1.02–1.34); arthritis 1.28 (1.11–1.47); asthma 1.67 (1.33–2.10); diabetes 1.51 (1.24–1.85); hearing problem 1.69 (1.36–2.11); tuberculosis 1.57 (1.09–2.25); visual impairment 2.01 (1.40–2.88); and multimorbidity 1.38 (1.19–1.60).

**Table 3:** Associations between chronic conditions or multimorbidity and low physical activity (outcome) estimated by multivariable logistic regression

	Overall		Age 18–34 years		Age 35–49 years		Age 50–64 years		Age ≥65 years	
	OR [95% CI]	*P*-value	OR [95% CI]	*P*-value	OR [95% CI]	*P*-value	OR [95% CI]	*P*-value	OR [95% CI]	*P*-value
Angina	1.04	0.2736	0.84*	0.0133	1.00	0.9658	1.17*	0.0246	1.23**	0.0085
	[0.97,1.11]		[0.73,0.96]		[0.87,1.14]		[1.02,1.34]		[1.06,1.44]	
Arthritis	1.12*	0.0151	0.97	0.7811	1.07	0.3390	1.28***	0.0008	1.09	0.2756
	[1.02,1.23]		[0.76,1.22]		[0.93,1.24]		[1.11,1.47]		[0.94,1.26]	
Asthma	1.19**	0.0010	0.88	0.2187	1.04	0.7321	1.67***	<0.0001	1.38**	0.0057
	[1.07,1.32]		[0.72,1.08]		[0.85,1.26]		[1.33,2.10]		[1.10,1.73]	
Chronic back pain	1.00	0.9958	0.90	0.2926	1.03	0.7357	0.99	0.9550	0.97	0.7713
	[0.91,1.10]		[0.74,1.10]		[0.86,1.24]		[0.82,1.20]		[0.78,1.21]	
Diabetes	1.33***	<0.0001	1.20	0.3842	1.14	0.4057	1.51***	0.0001	1.57***	0.0001
	[1.17,1.50]		[0.79,1.83]		[0.84,1.54]		[1.24,1.85]		[1.24,1.98]	
Edentulism	1.46***	<0.0001	1.24	0.1568	1.37*	0.0159	1.20	0.0758	1.22*	0.0197
	[1.30,1.64]		[0.92,1.68]		[1.06,1.76]		[0.98,1.46]		[1.03,1.45]	
hearing problem	1.90***	<0.0001	1.54	0.0549	1.49	0.0632	1.69***	<0.0001	1.35**	0.0034
	[1.66,2.18]		[0.99,2.39]		[0.98,2.27]		[1.36,2.11]		[1.10,1.65]	
Tuberculosis	1.24*	0.0304	0.96	0.8179	1.37	0.0876	1.57*	0.0151	1.12	0.6632
	[1.02,1.51]		[0.69,1.33]		[0.95,1.97]		[1.09,2.25]		[0.67,1.89]	
Visual impairment	2.29***	<0.0001	0.91	0.8194	2.24**	0.0020	2.01***	0.0002	2.04***	0.0001
	[1.83,2.85]		[0.42,1.99]		[1.34,3.75]		[1.40,2.88]		[1.44,2.89]	
Multimorbidity	1.31***	<0.0001	0.81*	0.0344	1.17*	0.0448	1.38***	<0.0001	1.37***	<0.0001
	[1.21,1.42]		[0.67,0.98]		[1.00,1.37]		[1.19,1.60]		[1.18,1.59]	

All models are adjusted for age, sex, wealth, education and country

The total amount of moderate to vigorous physical activity over the last week was calculated and those scoring <150 min were considered to have low physical activity

Multimorbidity refers to two or more chronic conditions

Abbreviation: *OR* Odds Ratio, *CI* Confidence Interval

*p < 0.05, **p < 0.01, ***p < 0.001

The results of the mediation analysis among those aged ≥50 years are shown in Table 4. The indirect effect was significant in all analyses. For the individual chronic conditions, depression mediated 5.1% (visual impairment) to 23.5% (angina) of the association between the chronic condition and low physical activity. Mobility difficulties explained more than 25% of the association for seven out of the eight chronic conditions with particularly high mediated percentages observed for angina (95.3%) and arthritis (67.8%). Pain was a major mediator for angina (65.9%) and arthritis (64.9%), and sleep problems mediated between 5.9% (edentulism) to 43.7% (angina) of the association. In terms of multimorbidity, depression, mobility difficulties, pain and sleep mediated 12.5, 56.4, 38.7 and 21.6% of the association respectively.

**Table 4:** Depression and other health status as mediators in the association between chronic conditions and low physical activity among older adults (aged ≥50 years)

		Total effect		Direct effect		Indirect effect		
Exposure	Mediator	OR [95% CI]	*P*-value	OR [95% CI]	*P*-value	OR [95% CI]	*P*-value	% Mediated
Angina	Depression	1.17 [1.05–1.29]	0.0040	1.12 [1.01–1.25]	0.0283	1.04 [1.02–1.05]	<0.0001	23.5
	Mobility	1.16 [1.05–1.28]	0.0050	1.01 [0.91–1.12]	0.8970	1.15 [1.12–1.18]	<0.0001	95.3
	Pain/discomfort	1.18 [1.06–1.30]	0.0015	1.06 [0.96–1.17]	0.2790	1.11 [1.08–1.15]	<0.0001	65.9
	Sleep/energy	1.17 [1.06–1.30]	0.0021	1.09 [0.99–1.21]	0.0868	1.07 [1.05–1.10]	<0.0001	43.7
Arthritis	Depression	1.19 [1.07–1.33]	0.0018	1.16 [1.04–1.30]	0.0067	1.02 [1.01–1.04]	<0.0001	13.7
	Mobility	1.20 [1.07–1.34]	0.0016	1.06 [0.94–1.19]	0.3286	1.13 [1.10–1.16]	<0.0001	67.8
	Pain/discomfort	1.18 [1.06–1.32]	0.0027	1.06 [0.95–1.19]	0.3142	1.12 [1.08–1.15]	<0.0001	64.9
	Sleep/energy	1.18 [1.06–1.32]	0.0024	1.13 [1.01–1.26]	0.0377	1.05 [1.03–1.07]	<0.0001	30.2
Asthma	Depression	1.55 [1.29–1.85]	<0.0001	1.50 [1.25–1.79]	<0.0001	1.03 [1.02–1.05]	0.0002	7.6
	Mobility	1.52 [1.29–1.78]	<0.0001	1.34 [1.14–1.57]	0.0004	1.13 [1.10–1.17]	<0.0001	30.3
	Pain/discomfort	1.55 [1.31–1.84]	<0.0001	1.44 [1.22–1.70]	<0.0001	1.08 [1.05–1.11]	<0.0001	17.3
	Sleep/energy	1.56 [1.31–1.86]	<0.0001	1.48 [1.24–1.76]	<0.0001	1.05 [1.03–1.08]	<0.0001	11.8
Diabetes	Depression	1.48 [1.27–1.74]	<0.0001	1.45 [1.24–1.69]	<0.0001	1.03 [1.01–1.04]	0.0004	6.8
	Mobility	1.54 [1.32–1.80]	<0.0001	1.35 [1.15–1.57]	0.0002	1.14 [1.11–1.18]	<0.0001	31.2
	Pain/discomfort	1.51 [1.29–1.76]	<0.0001	1.40 [1.20–1.64]	<0.0001	1.08 [1.05–1.10]	<0.0001	17.6
	Sleep/energy	1.51 [1.29–1.76]	<0.0001	1.44 [1.24–1.68]	<0.0001	1.05 [1.03–1.07]	<0.0001	11.2
Edentulism	Depression	1.20 [1.04–1.40]	0.0143	1.19 [1.03–1.38]	0.0217	1.01 [1.00–1.02]	0.0110	6.7
	Mobility	1.25 [1.07–1.46]	0.0041	1.23 [1.05–1.43]	0.0090	1.02 [1.00–1.04]	0.0281	8.7
	Pain/discomfort	1.20 [1.04–1.39]	0.0142	1.18 [1.02–1.37]	0.0264	1.02 [1.01–1.03]	0.0051	9.2
	Sleep/energy	1.20 [1.04–1.39]	0.0137	1.19 [1.03–1.38]	0.0204	1.01 [1.00–1.02]	0.0141	5.9
Hearing problem	Depression	1.43 [1.22–1.67]	<0.0001	1.39 [1.19–1.64]	<0.0001	1.02 [1.01–1.04]	0.0005	6.3
	Mobility	1.52 [1.30–1.78]	<0.0001	1.37 [1.17–1.60]	0.0001	1.11 [1.08–1.14]	<0.0001	25.6
	Pain/discomfort	1.52 [1.31–1.76]	<0.0001	1.42 [1.23–1.65]	<0.0001	1.07 [1.04–1.09]	<0.0001	15.5
	Sleep/energy	1.47 [1.26–1.73]	<0.0001	1.42 [1.21–1.66]	<0.0001	1.04 [1.02–1.06]	<0.0001	10.0
Tuberculosis	Depression	1.35 [0.98–1.86]	0.0689	1.27 [0.92–1.76]	0.1480	1.06 [1.03–1.10]	0.0008	20.2
	Mobility	1.37 [0.99–1.88]	0.0553	1.21 [0.88–1.66]	0.2472	1.13 [1.07–1.20]	<0.0001	39.7
	Pain/discomfort	1.39 [1.01–1.90]	0.0412	1.28 [0.93–1.75]	0.1283	1.09 [1.05–1.13]	<0.0001	25.4
	Sleep/energy	1.40 [1.01–1.92]	0.0409	1.32 [0.96–1.81]	0.0929	1.06 [1.03–1.09]	0.0001	17.6
Visual impairment	Depression	2.12 [1.61–2.77]	<0.0001	2.04 [1.55–2.67]	<0.0001	1.04 [1.02–1.06]	0.0006	5.1
	Mobility	2.10 [1.60–2.77]	<0.0001	1.67 [1.27–2.20]	0.0002	1.26 [1.20–1.32]	<0.0001	30.9
	Pain/discomfort	2.10 [1.61–2.75]	<0.0001	1.84 [1.41–2.40]	<0.0001	1.14 [1.10–1.19]	<0.0001	17.9
	Sleep/energy	2.12 [1.62–2.77]	<0.0001	1.92 [1.47–2.50]	<0.0001	1.11 [1.07–1.15]	<0.0001	13.4
Multimorbidity	Depression	1.37 [1.23–1.54]	<0.0001	1.32 [1.18–1.48]	<0.0001	1.04 [1.02–1.06]	<0.0001	12.5
	Mobility	1.36 [1.22–1.52]	<0.0001	1.14 [1.02–1.29]	0.0236	1.19 [1.15–1.23]	<0.0001	56.4
	Pain/discomfort	1.38 [1.24–1.54]	<0.0001	1.22 [1.09–1.36]	0.0006	1.13 [1.09–1.18]	<0.0001	38.7
	Sleep/energy	1.37 [1.23–1.53]	<0.0001	1.28 [1.15–1.43]	<0.0001	1.07 [1.04–1.10]	<0.0001	21.6

Models are adjusted for age, sex, education, wealth and country

The total amount of moderate to vigorous physical activity over the last week was calculated and those scoring <150 min were considered to have low physical activity

Multimorbidity refers to two or more chronic conditions. Depression = presence in the past 12 months (DSM-IV). Mobility problems, pain/discomfort and sleep/energy problems = presence in the past 30 days; each item was scored on a five-point scale ranging from ‘none’ to ‘extreme/cannot do’. For each separate domain, we used a factor analysis to obtain a factor score which was later converted to scores ranging from 0 to 100

Abbreviation: *OR* Odds Ratio, *CI* Confidence Interval

## Discussion

### General findings

To the best of our knowledge, the current study is the first large-scale (*n* = 228,024), multinational (46 LMICs) analysis investigating chronic conditions, multimorbidity and low levels of physical activity. We found that most chronic conditions were associated with low physical activity in the overall sample, although this relationship was most notable among the older population. Our mediational analysis for the older age group showed that mobility difficulties was an important factor for most of the chronic conditions studied, while pain was a central factor for angina and arthritis. Depression and sleep problems also explained a large proportion of these associations, particularly for angina. As for multimorbidity, mobility difficulties and pain were important factors mediating low physical activity. The identification of mediators offers potential targets for future public health interventions and our study provides important information. For example, in the overall sample, those with arthritis were less likely to achieve the physical activity recommendations (OR = 1.12). This significant association was mediated by depressive feelings, pain, mobility and sleep problems. Arthritis is associated with pain which might cause mobility and sleep problems and ultimately feelings of depression, which in turn will be a barrier for physical activity participation [45]. The current data indicate that similar vicious cycles may also exist for other chronic conditions and multimorbidity.

Although there is no rigorous evidence that physical activity has a direct effect on the pathogenesis of arthritis [21], there is evidence that physical activity reduces pain [46] and depression [47] and improves sleep [48] and functionality [49] in people with arthritis. In terms of the training effect on mobility impairments, the immediate mechanism of action may be through improved balance, muscle strength and endurance [21]. If there is any sign of acute joint inflammation and/or a worsening of symptoms, the affected joint should rest until a drug treatment has taken effect [21]. Also the nature of the training can be varied to include, for example, aquatic exercises [50], although this will not always be possible in most low resourced settings. A similar line of action can be proposed for people with tuberculosis. Low physical activity in people with tuberculosis is mainly mediated through infection-related anemia and is associated with elevated acute phase response [51]. In the acute stages, pharmacotherapy is primordial [52]. To the best of our knowledge, there is no evidence for the beneficial effects of physical activity on the management of tuberculosis, and tuberculosis should rather be considered a barrier for physical activity participation. However, it could be hypothesized that physical activity might improve conditions associated with tuberculosis such as pain, depression and functional limitations, although research to confirm this is needed. In contrast, there is rigorous evidence for the beneficial effects of physical activity in chronic conditions such as diabetes, angina and low chronic back pain [21]. Health care professionals need to consider barriers, such as pain, sleep and mobility problems, in addition to condition-specific contra-indications.

In people with diabetes, physical activity interventions should be delayed until acute high blood sugar levels have been corrected [21]. Low-cost methods to assess blood sugar levels in low resourced settings need to be developed. In the case of active proliferative retinopathy, it is recommended that high-intensity training or training involving Valsalva maneuvers be avoided [21]. Strength training should be done with light weights and at low contraction velocity. In the case of neuropathy and the risk of foot ulcers, body-bearing activities should be avoided as repeated strain on neuropathic feet can lead to ulcers and fracture. Non-body-bearing exercise is recommendable such as cycling and swimming [21], but other strategies for resource-limited settings should be explored. Physical activity is not a contra-indication for stable (at least 5 days) angina [53] nor for chronic back pain [21] or asthma [21]. Effectiveness studies are needed to explore how international guidelines for these chronic conditions such as 12 weeks supervised training with individually organized training programs after an initial exercise test: two to five sessions a week of 30–60-min at an intensity of 50–80% of the maximum exercise capacity and/or daily low-intensity training walking over 30 min, increasing over time under the supervision of the rehabilitation team [21], can be implemented in resource-limited settings.

We also found significant associations between low physical activity and hearing problems and visual impairments. Hearing problems [54] and visual impairments [55] should therefore be considered as an important barrier for being physically active in LMICs. Stigma and discrimination associated with these chronic conditions and a lack of social support may further complicate physical activity participation in these populations. In the same way, negative self-perceptions associated with edentulism might, particularly in younger patients, be a barrier for participation in physical activity [56].

### Practical implications and future research

The key to management of chronic conditions and multimorbidity is to strengthen a multidisciplinary approach simultaneously targeting both lifestyle factors and physical health outcomes (e.g., risk for chronic diseases, multimorbidity). In LMICs, in addition to economic restraints, other challenges may also undermine the development of effective and sustainable primary and secondary interventions. For example, implementing non-pharmacological interventions may be difficult in LMICs, due to the predominant biomedical model of practice within existing healthcare systems [57]. Results from a qualitative study that explored treatment adherence among patients with diabetes, hypertension or both, in a South-African community, suggest that factors that may influence adherence to behavior change interventions may be multifactorial, including the attribution of the origin of the illness, negative experiences with the public healthcare system, financial problems, transport problems and lack of social support [58]. Our analyses show that strategies to deal with lifestyle factors, such as physical inactivity, are urgently needed in LMICs, particularly targeting the earlier stages of disease.

First of all, there is a clear need to increase awareness of the importance of considering physical activity participation among care providers in LMICs. Continued medical education should be used to inform care providers on the importance of assessing physical activity levels and how cognitive behavioral principles (e.g., goal setting, problem-solving etc.) can be employed to assist patients to increase physical activity levels. We propose a dual strategy of developing both a smaller group of master trainers/supervisors (e.g., exercise physiologists and physiotherapists) and researchers and a larger group of practitioners (e.g., nurses) trained in the basics of cognitive behavioral physical activity strategies. This method has been successfully employed for cognitive behavioral therapy in trials in LMICs [59, 60]. A stepped-care approach, where patients start with self-management, may be a feasible strategy in LMIC settings. Then, if patients do not achieve guideline- specific levels of physical activity, they could continue with a manualized approach under the supervision of a non-specialist worker (e.g., nurses, occupational therapists). Patients would only be referred to a specialist supervisor (e.g., exercise physiologists and physiotherapists) if no significant increase in physical activity levels occurs, for example due to pain, sleep problems or mobility problems. It is known that inclusion of exercise physiologists or physiotherapists reduces drop out rates from physical activity interventions and consequently improves outcomes [61]. Careful consideration of what physical activity implementation strategies would be most efficacious, and evaluation of this stepped-care approach, is essential for chronic conditions. The current available evidence is, however, solely based on evidence from high-income countries. Efficacy trials of physical activity interventions among people with chronic conditions in different cultural settings across LMICs are urgently needed. In addition, effectiveness trials in diverse cultural settings could explore whether assisting people in fulfilling the following three universal and psychological needs will increase the likelihood that they adopt or maintain the prescribed recommendations/interventions of physical activity: (a) the need for autonomy (i.e., experiencing a sense of psychological freedom when engaging in physical activity), (b) the need for competence (i.e., ability to attain desired outcomes following the physical activity program), and (c) the need for relatedness (i.e., being socially connected when being physically active). If the efficacy and effectiveness of physical activity interventions are well established in better equipped scientific settings with research staff trained in physical activity prescription, the final step will be to fund interventions and initiatives to translate research findings into “real-world” settings while exploring its cost-effectiveness. In order to justify the inclusion of physical activity programs as a routine component in the treatment of chronic diseases and multimorbidity in LMICs, cost-benefit analyses should be conducted in order to quantify the financial implications of diverting resources or investing funds into such initiatives. Therefore, next to intervention studies exploring the efficacy of physical activity programs, effectiveness research capable of driving practice change, along with policy-level research, is urgently required. Ministries of health and education will play a critical role in this governance and policy development step.

If research shows that physical activity is efficacious and effective in the prevention and management of chronic diseases in LMICs, physical activity should be mainstreamed in existing health systems at all levels of care. Governments of LMICs will need to provide appropriate environments for physical activity including, space, infrastructure and tools. Such affirmative action is already required. Governmental and non-governmental agencies need to increase public health awareness of the importance of physical activity in people with chronic conditions. For example, physical activity should be integrated into the existing Information, Education and Communication public health awareness programs of the World Health Organization. Targeted messages should be developed in order to make these campaigns affordable. The benefits of engaging in physical activity should be properly outlined; any fears and wrongly held beliefs within various cultural contexts dispelled and, appropriate initiations steps (e.g., starting at a low intensity) and methods to maintain an active lifestyle should be included in community awareness programs.

### Limitations and strengths

These findings should be interpreted in light of some limitations. First, the study is cross-sectional and cause and effect cannot be deduced. Therefore, it remains unclear whether lack of physical activity was caused by chronic conditions or vice versa. For example, physical inactivity is known to be a risk factor for cardiovascular diseases [62], while pain caused by arthritis or angina may limit the ability of an individual to engage in physical activity. Second, whilst we included all physical health conditions which were assessed within the WHS, other physical conditions such as stroke and hypertension which are frequently reported in multimorbidity indices [63] may have been present and not identified in the study. Third, since the information on chronic conditions and physical activity was based on self-report, reporting biases may exist. For example, across the entire sample only 29.2% were classified as being insufficiently active, which is lower than expected based on previous research. Therefore, the relationship between multimorbidity and low physical activity in our study may have been underestimated. Fourth, by separating the sample into dichotomous categories of sufficient and insufficient physical activity, we were not able to examine how different quantities of physical activity may affect morbidity. For instance, it is possible that achieving significantly more than 150 min confers additional benefits beyond simply meeting the minimum guidelines. Similarly, those who are almost completely inactive may have significantly more risk of chronic conditions than those who just fall short of the recommended 150 min. Future research to establish the optimal amounts of physical activity to reduce the risk of chronic illness would be useful to inform physical activity policy and practice guidelines. Fifth, the present study did not include institutionalized people, which may limit generalizability at a national level. Finally, we allowed for one missing variable when calculating the total number of chronic conditions. This was done to retain a larger sample size but some level of misclassification may exist. Nonetheless, the strengths of the study include the multi-national scope focused on LMICs, countries which are under-represented in the prior research literature. Additionally, we clarified numerous important mediators that can be targeted for future physical activity interventions.

## Conclusions

The current study demonstrates that individuals with chronic conditions and multimorbidity are significantly more likely to engage in low levels of physical activity. Research on the efficacy and effectiveness of physical activity in management of chronic diseases and multimorbidity in LMICs should be a priority for funding bodies. When an evidence base is built, physical activity needs to be mainstreamed in existing health policies and strategies at all levels of care. To this end, policy makers and budget holders will need to invest in physical activity as part of a multidisciplinary treatment package for a wide range of chronic conditions, while health professionals will need to improve their physical activity assessment and prescriptions skills. Physiotherapists and exercise physiologists might assist in complex cases where pain, mobility difficulties, sleep problems and depression are important barriers for initiating or adopting an active lifestyle. Finally, (inter) national agencies will do well to increase public health awareness of the importance of physical activity in people with chronic diseases and multimorbidity in LMICs.

## Additional file

Additional file 1: Table S1. Questions used to assess health status. (DOCX 11 kb)

## Abbreviations

CI: Confidence interval
LMICs: Low- and middle-income countries
OR: Odds ratio
WHS: World Health Survey
