# Supplementary material for: Chronic physical conditions, multimorbidity and physical activity across 46 low- and middle-income countries
Source: Int J Behav Nutr Phys Act. 2017 Jan 18;14:6. doi: 10.1186/s12966-017-0463-5 (PMC5241915; doi:10.1186/s12966-017-0463-5)
Supplement: Additional file 1: Table S1. — Questions used to assess health status. (DOCX 11 kb) [file 12966_2017_463_MOESM1_ESM.docx]

| **Additional file 1: Table S1** Questions used to assess health status | |
| --- | --- |
| **Mobility** | [1] Overall in the last 30 days, how much difficulty did you have with moving around? |
|  | [2] In the last 30 days, how much difficulty did you have in vigorous activities, such as running 3 km (or equivalent) or cycling? |
| **Pain and discomfort** | [1] Overall in the last 30 days, how much of bodily aches or pains did you have?  [2] In the last 30 days, how much bodily discomfort did you have? |
| **Sleep and energy** | [1] Overall in the last 30 days, how much of a problem did you have with sleeping, such as falling asleep, waking up frequently during the night or waking up too early in the morning? |
|  | [2] In the last 30 days, how much of a problem did you have due to not feeling rested and refreshed during the day (e.g. feeling tired, not having energy)? |
